# Evaluation of post-mortem lateral cerebral ventricle changes using sequential scans during post-mortem computed tomography

**DOI:** 10.1007/s00414-016-1327-2

**Published:** 2016-04-05

**Authors:** Iwao Hasegawa, Akinobu Shimizu, Atsushi Saito, Hideto Suzuki, Hermann Vogel, Klaus Püschel, Axel Heinemann

**Affiliations:** 1 Department of Legal Medicine, University Medical Center Hamburg-Eppendorf, Butenfeld 34, Hamburg, 22529 Germany; 2 Department of Forensic Medicine, School of Medicine, Tokai University, Shimokasuya 143, Isehara, Kanagawa 259-1193 Japan; 3 Tokyo Medical Examiner’s Office, Otsuka 4-21-18, Bunkyo, Tokyo, 112-0012 Japan; 4 Institute of Engineering, Tokyo University of Agriculture and Technology, Naka-cho 2-24-16, Koganei, Tokyo 184-0012 Japan

**Keywords:** Post-mortem computed tomography, Sequential scan, Post-mortem change, Lateral cerebral ventricle, Post mortem interval

## Abstract

In the present study, we evaluated post-mortem lateral cerebral ventricle (LCV) changes using computed tomography (CT). Subsequent periodical CT scans termed “sequential scans” were obtained for three cadavers. The first scan was performed immediately after the body was transferred from the emergency room to the institute of legal medicine. Sequential scans were obtained and evaluated for 24 h at maximum. The time of death had been determined in the emergency room. The sequential scans enabled us to observe periodical post-mortem changes in CT images. The series of continuous LCV images obtained up to 24 h (two cases)/16 h (1 case) after death was evaluated. The average Hounsfield units (HU) within the LCVs progressively increased, and LCV volume progressively decreased over time. The HU in the cerebrospinal fluid (CSF) increased at an individual rate proportional to the post-mortem interval (PMI). Thus, an early longitudinal radiodensity change in the CSF could be potential indicator of post-mortem interval (PMI). Sequential imaging scans reveal post-mortem changes in the CSF space which may reflect post-mortem brain alterations. Further studies are needed to evaluate the proposed CSF change markers in correlation with other validated PMI indicators.

**Electronic supplementary material:**

The online version of this article (doi:10.1007/s00414-016-1327-2) contains supplementary material, which is available to authorized users.

## Introduction

In forensic practice, post-mortem computed tomography (PMCT) provides important information prior to an autopsy [1–8]. PMCT can detect gasses within the body [9, 10] in addition to certain abnormalities (e.g., bone fractures [11, 12]). In some cases, it is difficult to differentiate post-mortem change from organic abnormalities [13–17]. For example, pneumonia and post-mortem liquid sedimentation produce similar CT images [18, 19]. Furthermore, disease-oriented pathological brain edema and post-mortem cerebral change also appear similar on CT scans [20]. For practical applications of PMCT, it is important to differentiate disease-oriented pathological change from post-mortem change. However, systematic studies evaluating post-mortem change in PMCT are rarely focusing on longitudinal post-mortem observations[21–25], particularly for post-mortem brain alterations [20, 26]. To address this issue, the current study included an assessment of post-mortem change in PMCT, which will be referred to as a “sequential scan project.” This sequential scan paradigm includes successive, periodical CT scans of the same post-mortem subject. This method enables to compare the same image slices at different post-mortem time points. During the early post-mortem phase, muscle rigidity and skin lividity increase and body temperature decreases; these changes are readily observable using conventional, external forensic inspection. However, all organs inside the body also undergo some amount of post-mortem change [16, 27]. During an autopsy, it is possible to determine organ condition at a certain time post-mortem. However, an autopsy is a relatively destructive inspection, and it is difficult to observe the temporal course of post-mortem change inside the body using a conventional autopsy. Using a sequential scan procedure, it is possible to observe the unfolding of post-mortem change inside the whole body, without a destructive inspection. Post-mortem brain alterations including CSF volume changes have been described [3, 20, 26, 28], but there is still a need for clarification of the extent of these phenomena in comparison to antemortem brain edema as some authors denied brain swelling in the very early PMI [20]. The LCV volumes have recently been described as weak indicators of ante mortem cerebral edema [28] but their post mortem alteration had not been exactly deliminated in that retrospective study. Being inconclusive as marker of antemortem brain edema (probably benefitting from minor impact of the cause of death on their volume change as compared to the narrowing of temporal horns and symmetrical herniation of the cerebellar tonsils), the LCV volume attracts interest regarding its sensitivity for time-dependent cadaveric modifications.

Another important issue is the Hounsfield units (HU) attenuation change which has been described for gray and white matter of the brain [16, 26] but not for the CSF in the lateral ventricles and particularly for its implications regarding PMI estimations. For the current study, the lateral cerebral ventricles (LCVs) were evaluated using this sequential scan method, because they offer the most convenient measurement of HU radiodensity in comparison to other CSF spaces as unwanted inclusion of boundaries with the risk of partial volume artifacts can be minimized.

## Materials and methods

Three deceased individuals from the emergency room at the University Medical Center who were diagnosed with ischemic heart disease served as subjects for the present study. Certified clinical physicians determined time of death. After each subject was transferred to the department of legal medicine, the first PMCT was performed between 1 and 2 h post-mortem. Scan times are shown in Table 1. Each subject was placed in a supine position on a horizontal CT table. A full-body, multi-slice, PMCT procedure included the following parameters: 130 kV, 350 mA, 1.3-mm scan thickness, 1.5 pitch with a 6-channel multi-detector CT (MX8000, Philips, Amsterdam, the Netherlands). Images were reconstructed and analyzed using OsiriX open-source imaging software (Version 3.9 64 bits, OsiriX Foundation, Geneva, Switzerland) on a Mac OS X (Version 10.5.4, Apple Inc., CA, USA). The temperature of the CT room was kept at 5 °C using an air conditioner and natural, cold air. After the first PMCT, to obtain complete reproducible cross sectional images, the subject was not moved until the last scan session (16–24 h post-mortem). For the cerebral scans, all cross-sectional images were reconstructed to the plane parallel to the OM (orbitomeatal) line. After the sequential scans, forensic practitioners examined the subjects; this examination included a conventional autopsy, and a cause of death was determined. To eliminate the possibility of cerebral organic diseases and other abnormalities, subjects’ brains were evaluated. No specific organic cerebral disease or damage was present in the three subjects. Subject details can be found in Table 1.

**Table:** Table 1

	Age	Sex	Scan time, hours post-mortem	Cause of death
Case #1	59 y/o	male	2–20 (every 1 h)	Ischemic heart disease
Case #2	73 y/o	male	1–16 (every 1 h)	Ischemic heart disease
Case #3	71 y/o	male	1, 2, 3, 5, 6, 9, 12, 18, 24	Ischemic heart disease

Cerebral CT images were evaluated on the horizontal plane in three dimensions. The average HU in both LCVs was monitored using a graph cut-based segmentation algorithm [29–33]. The measurement was performed automatically after extraction of the region of the lateral cerebral ventricle from a three-dimensional (3D) CT image using the semi-automated segmentation algorithm. This algorithm is based on graph-cuts, which is one of the most popular segmentation algorithms in the field of medical image processing [30, 31], and the entire procedure is shown in Fig. 2. Seed regions in step 1 were generated using the region of the lateral cerebral ventricle roughly delineated using a computer mouse, followed by shape smoothing by morphological operations, or dilation and erosion [33]. To generate seed regions for the remaining 3D CT images with different acquisition times for the same cadaver, a free-form deformation was employed [32]. LCV volume and HU were also measured automatically by counting the number of voxels within the LSV and computing the average HU within the LSV.

The present study was performed with informed consent provided by the participants, according to established laws and ethical guidelines within the University Medical Center Hamburg-Eppendorf in Germany. The authors have no conflicts of interest directly relevant to the content of this article.

## Results

**Fig. 1:**
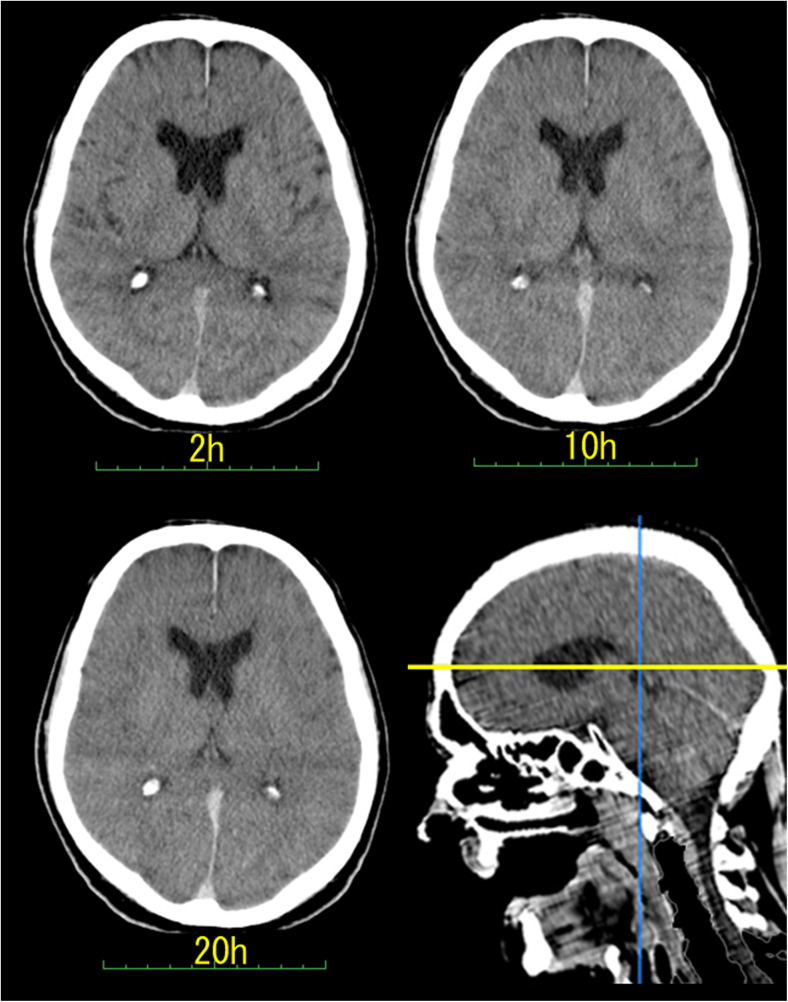
Case #1. The *number under each image* denotes post-mortem time. The *right-bottom image* denotes the sagittal plane from which these slices were obtained. The *yellow line* indicates the OM line, 3 cm above the highest point of the posterior clinoid process

**Fig. 2:**
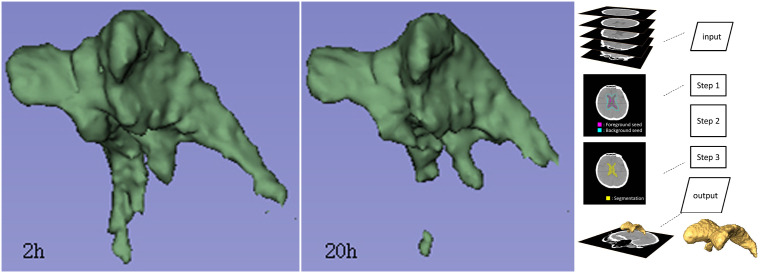
Case #1. Three-dimensional image of the lateral cerebral ventricle (LCV). Left : 2 h post-mortem. The volume was 26.8 ml. Middle: 20 h post-mortem. The volume was 21.8 ml. The LCV was slightly thinner at 20 h than at 2 h. Right: Flowchart of semi-automated segmentation algorithm of the LCV. Input: 3D CT images of a cadaver. Step 1: Input of seed regions of the LCV and background by an observer for a 3D CT image. The seed regions are deformed to generate seed regions for the remaining 3D CT images with different acquisition times in the same cadaver. Step 2: Statistical parameter estimation of a probability distribution for each 3D CT image by using an expectation maximization algorithm followed by likelihood computation. Step 3: Graph-cut segmentation for each 3D CT image that optimizes a given energy function. Output: 3D-labeled images of the LCV in the 3D CT images, in which the gray values inside the LCV are one and the outside is zero

**Fig. 3:**
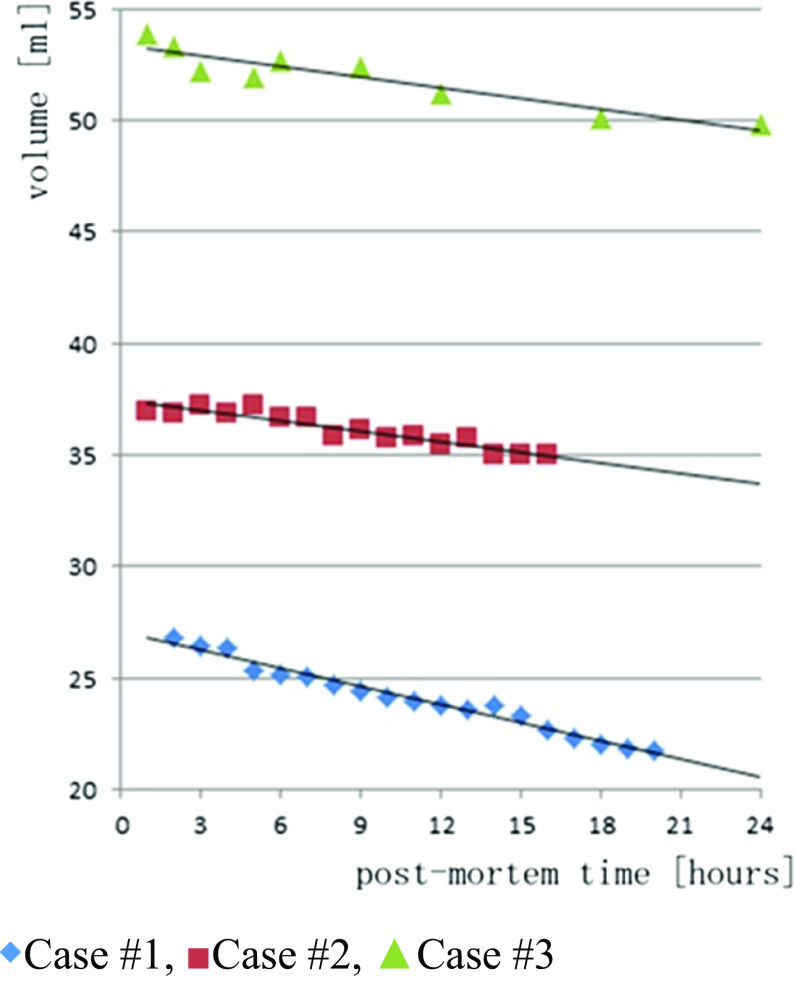
LCV volume decreases as a function of post-mortem time

**Fig. 4:**
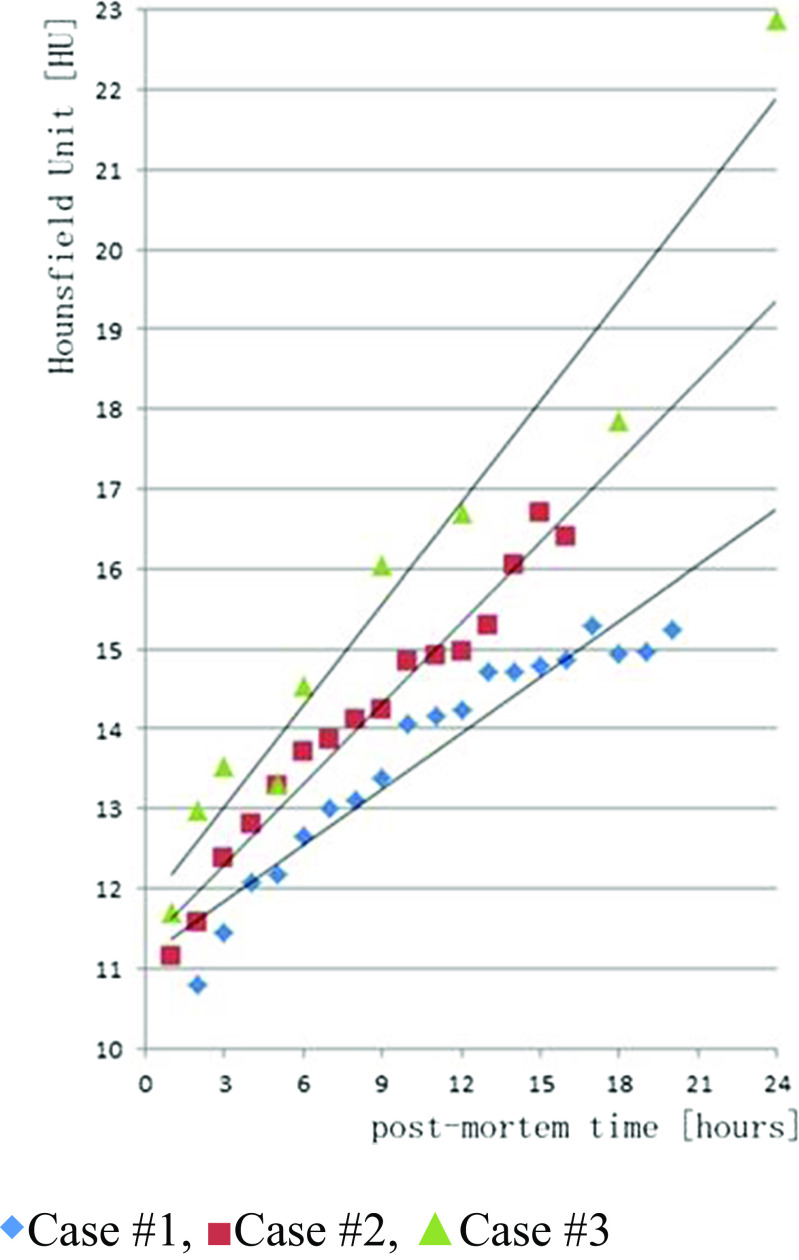
HU unit increases within the LCVs as a function of post-mortem time

In total, three CT image series (one series per subject) were obtained. The CT axial plane images obtained from Case #1 are shown in Fig. 1. As shown in the figure, the size of the frontal horns within the LCVs decreased over time. A 3D image of Case #1’s LCVs from 2–24 hours post-mortem is shown in Fig. 2. From this figure, the diminished size of the posterior horns within the LCVs can be seen. For all three subjects, the relationship between post-mortem time and LCV volume is shown in Fig. 3. With regard to Case #1, it was not possible to measure precise LCV volume after 21 h post-mortem because of excessive LCV narrowing. There was a robust, linear decrease in LCV volume as a function of post-mortem time. The relationship between post-mortem time and HU is shown in Fig. 4. The HU attenuation gradually increased over time. The linear equation formulae for these three cases are shown in Table 2.

**Table 2:** Post-mortem time change formulae for the lateral cerebral ventricles

	Volume	±SE	*R* ^2^	Hounsfield unit	±SE	*R* ^2^
Case #1	y = −0.27 × + 27.08	±0.62	0.98	y = 0.23 × + 11.12	±0.33	0.92
Case #2	y = −0.16 × + 37.45	±0.20	0.89	y = 0.34 × + 11.28	±0.41	0.97
Case #3	y = −0.16 × + 53.39	±0.51	0.87	y = 0.42 × + 11.74	±1.13	0.95

*SE* standard error, *R* ^2^ coefficient of determination

## Discussion

PMCT has become popular among forensic practitioners in recent years and is a useful method for estimating and investigating cause of death before conducting a conventional autopsy [34, 35]. In a traumatic case, PMCT is more sensitive than forensic autopsy in detecting skeletal injuries [5, 11, 36]. Furthermore, major cerebral bleeding is easy to recognize using PMCT [37]; in some cases, cerebral infarction can also be detected [38]. Thus, PMCT is a useful method for supporting conventional autopsy when detecting pathological brain lesions, particularly antemortem brain edema [28]. Post-mortem MRI results may be even better for the diagnosis of thin subarachnoid hemorrhages [3, 39], micro-bleedings, and infarctions [40], but MRI is less available for post-mortem routine procedures as well as cadaveric sequential studies. Thus, there is a need to estimate post-mortem changes in PMCT which is in routine use in a rising number of forensic departments. During post-mortem imaging, the brain and other organs are in a non-homeostatic condition which is to some extent comparable to changes in stroke lesions.

For more than 100 years, clinical radiologists have compared abnormal lesions with normal images. PMCT has only been available for approximately 10 years; however, systematic research observing post-mortem changes via CT is rare. This prompted the present “sequential scan project” to obtain normal images during post-mortem scans. The present study revealed post-mortem changes in CT images that varied according to the range of post-mortem time. Factors influencing early post-mortem change in PMCT include resuscitation [41, 42], massive hemorrhaging [24, 43], hypothermia, [44] and infections [22], but they have not been described as confounding factors for brain alterations. It is important to consider these factors when assessing post-mortem changes. For the present study, we focused on a common cause of death, i.e., ischemic heart disease, and assessed brains with the absence of pathological lesions, e.g., cerebral bleeding which would have influenced post-mortem brain changes in ways different from ischemic heart disease.

For the current project, each subject was continually scanned, over a 24-h period. The present results showed that LCV volume decreased as a function of post-mortem time (Fig. 3). Continuous ventricular narrowing can be interpreted as a consequence of post-mortem brain swelling [26] and/or CSF leakage; Brain swelling in PMCT may be caused by vasogenic edema and hyperemia (sometimes delayed after resuscitation) in the agonal stage [45] and eventually in later stages by autolysis and brain softening [16]. In cases of non-sudden deaths, there is an agonal stage of certain length during which hypoxic reperfusion of the brain occurs which fuels vasogenic edema. The—in vivo—well-known cytotoxic edema [46] resulting in an inflow of extracellular water into the intracellular space was considered by other study groups [20] as being irrelevant for postmortem cerebral swelling because the pure water shift within the brain should not affect the resulting total brain volume; however, this may only be true in a volume model that does not differentiate between brain tissue and inner cerebrospinal fluid spaces; so we conclude that also cytotoxic edema could actually explain the CSF volume decrease in our study. Osmotic swelling may be another explanation [28].

Further studies should consider the volume of inner and outer CSF space at the onset of a sequential series—if possible within a 2-h period after death or—if available—a volume rendering in the antemortem CT. Dilated LCVs due to age-related brain atrophy or pathological hydrocephalus may be more difficult to assess for post-mortem change as their narrowing relative to their initial volume expected to be less pronounced.

The average HU within the LCVs increased as function of post-mortem time (Fig. 4). One plausible reason for this result relates to decomposition of CSF proteins and/or the ependymal layer. Another possibility concerns CSF condensation while a swelling brain exerts pressure on the ventricles leading to shrinkage (only the liquid component can penetrate the membrane between the LCVs and the brain). Finally, inflow of protein-like substances from the periventricular brain tissues to the CSF could account for the observed HU values. The present study is restricted to a rough description of the phenomenon but did not clarify the issue of causal relationships between PMI and decrease of ventricle volume in detail. . For example, radioisotopes could be studied for their usefulness for tracing CSF movement during post-mortem evaluation. If it becomes possible to obtain a CSF sample every hour, CSF components could then be analyzed.

As shown in Fig. 4, it may be possible to estimate the PMI based on average HU values within the LCVs. In healthy, living volunteers, the normal range of the HU in the LCVs is around 5 [47]. During the first hour post-mortem, the HU quickly elevates from approximately 6 to 12. For the present study, single linear formulae were adopted from 1 h until 24 h post-mortem; however, these plots could be drawn on an S-curve. Further investigations with larger samples are needed to address this issue. Eventually, a combined predictor of PMI could be evaluated by using CSF attenuation and LCV volume as predictors but the minor slope of the volume curves in our three cases indicates a weakness for time interval prediction accuracy as compared with the more pronounced changes of HU attenuation.

A minor elevation of the HU in the LCVs is not reflective of minor brain bleeding into the LCVs; rather, this result reflects a post-mortem change revealed by forensic radiology.

In the post-mortem imaging field, the sequential scan method can be used to visualize early continuous changes in organs. In case those ethical standards are not compatible with longitudinal studies on human deceased, more focus should be put on cadaveric animal models [48].

## Electronic Supplementary Material

ESM 1 (JPG 291 kb)
